# The oldest mid-oceanic carbonate buildup complex: Setting and lithofacies of the Vendian (Late Neoproterozoic) Baratal limestone in the Gorny Altai Mountains, Siberia

**Published:** 2004-09-01

**Authors:** Yuko Uchio, Yukio Isozaki, Tsutomu Ota, Atsushi Utsunomiya, Mikhail M. Buslov, Shigenori Maruyama

**Affiliations:** *) Dept. Earth and Planetary Science, Tokyo Institute of Technology, 2-12-1, O-okayama, Meguro, Tokyo 152-8551, Japan; **) Dept. Earth Science and Astronomy, University of Tokyo, 3-8-1, Komaba, Meguro, Tokyo 153-8902, Japan; ***) United Institute of Geology, Geophysics and Mineralogy, Siberian Branch of Russian Academy of Science, Novosibirsk 630090, Siberia, Russia

**Keywords:** Vendian, seamount, carbonate buildup, Altai, Siberia

## Abstract

The Baratal limestone in the Gorny Altai Mountains, southern Siberia, occurs as large allochthonous blocks within a Cambrian accretionary complex that developed around the Siberia craton. Before the final accretion to Siberia in the Cambrian, the terrigenous clastic-free Baratal limestone was deposited directly upon a basaltic basement with a geochemical signature identical to that of modern oceanic plateau. The Baratal limestone with 598 ± 25 Ma (Early Vendian) Pb-Pb isochron age consists of three distinct facies; 1) massive lime mudstone with ooids and stromatolites, 2) bedded lime mudstone, and 3) limestone conglomerate/breccia dominated by ooid-bearing lime mudstone clasts. The first represents a shallow marine environment on top of an ancient oceanic plateau, while the latter two represent the deeper slope to bottom-of-slope facies of a plateau. The Vendian Baratal limestone provides the oldest example of a reconstructed shallow marine carbonate buildup complex developed on a plateau/seamount in a mid-ocean.

## Introduction

The Vendian-Cambrian boundary (VCB; ca. 543 Ma1)) was a remarkable timing of global environmental change associated with biological reorganization involving the well-known Ediacaran extinction and Cambrian radiation.2)–4) Previous studies have analyzed litho-, bio-, and chemo-stratigraphy across the VCB of ancient continental shelf sequences in Russia, South China, Canada and Namibia, etc.5),6) However, contemporaneous paleo-environmental information from mid-ocean was not available simply because all the seafloors existing at that time have been lost by oceanic subduction. In modern oceans there is no rock formed on the ancient oceanic crust older than 200 Ma.

A new viewpoint and analytical method to examine pre-200 Ma oceanic environments has been developed through researches on deep-sea chert in Paleozoic and Mesozoic accretionary complexes in Japan.7) Numerous ancient oceanic rocks, such as deep-sea chert, mid-oceanic ridge basalt (MORB), and oceanic island basalt (OIB), have been accreted to continental margins by oceanic subduction, and their fragments occur as allochthonous blocks in ancient accretionary complexes. 8),9) Such exotic blocks of OIB-type basaltic green-stones frequently accompany shallow marine carbonates that are interpreted to have formed primarily on ancient seamounts in mid-oceanic domains,10),11) and they provide the sole source of information on paleoenvironment of lost oceans as first demonstrated in studies of the Permo-Triassic boundary event.12),13)

**Fig. 1:**
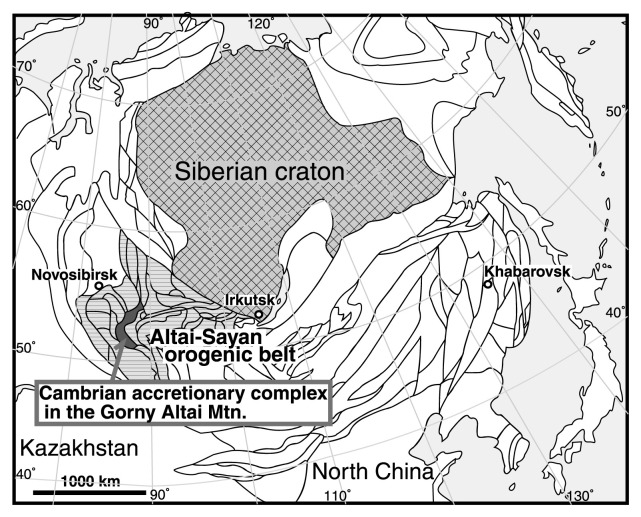
Index map of the study area in the Gorny Altai Mountains, southern Siberia, modified from Sengör and Natalin (1996).

The same approach appears promising for the VCB event, and therefore we have studied Vendian mid-oceanic limestone blocks enclosed in a Cambrian accretionary complex in the Gorny Altai Mountains of southern Siberia (Fig. 1). Our 5-year field research program since 1997 has produced a detailed analysis of the Cambrian accretionary complex and pre-accretion primary stratigraphy of the mid-oceanic seamount-related Baratal limestone. This paper describes the geology and stratigraphic framework of the Baratal limestone that represents the world’s oldest example of a reconstructed mid-oceanic carbonate buildups on a seamount.

## Altai-Sayan orogenic belt in the Gorny Altai Mountains

**Fig. 2:**
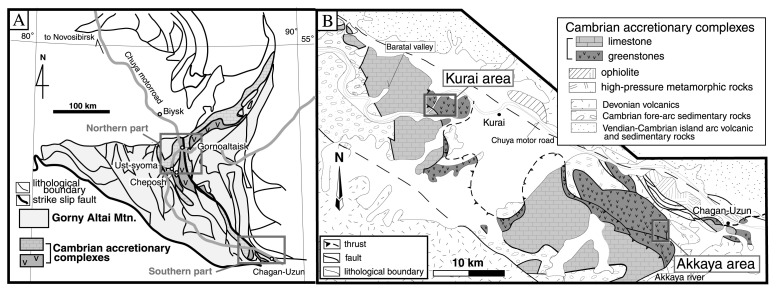
Geologic sketch map of the Gorny Altai Mountains (A) and lithologic map of the Cambrian accretionary complex in the Kurai and Akkaya areas (B) modified from Buslov *et al*. (1993).

The triangular-shaped Gorny Altai Mountains in southern Siberia form the central part of the Paleozoic-Mesozoic Altai-Sayan orogenic belt,14),15) that extends from Kazakhstan through southern Siberia to North China, ca. 1,500 km wide and ca. 5,000 km long along the southwestern margin of the Siberian craton (Figs. 1, 2A). Buslov *et al*. (1993) and Buslov & Watanabe (1996) extensively studied the Gorny Altai Mountains and first introduced practical plate-tectonic interpretations of the Paleozoic geology.16),17) They distinguished several geologic units including the Cambrian accretionary complex, Vendian to Cambrian high-P/T metamorphic rocks and ophiolite, Vendian to Devonian island arc-type igneous rocks, and Cambrian to Carboniferous fore-arc sedimentary rocks. All these units formed in a late Neoproterozoic to early Paleozoic tectonic framework, which involved subduction of oceanic plates beneath the Siberian craton.16),17)

The Cambrian accretionary complex occurs in a narrow belt in the central Gorny Altai Mountains (Fig. 2B). This complex comprises basaltic greenstones, limestone, and terrigenous clastics, and underwent greenschist to sub-greenschist facies regional metamorphism but no strong deformation. Early Cambrian Archaeocyathids occur in limestone clasts in conglomerates, and Early Cambrian sponge spicules in siliceous mudstone.16),18) These relations indicate that the accretionary complex formed in a peri-Siberian trench no earlier than the Early Cambrian. Accordingly, this unit is tentatively described as a Cambrian accretionary complex in this article.

The greenstones and limestones occur as allochthonous blocks in a matrix of younger clastic sediments. The allochthonous limestone, previously called the Baratal suite,19) contains Vendian-type stromatolites and microphytolites.16),19) Recently, Nohda *et al*. (2003) measured a bulk Pb-Pb isochron age of the Baratal limestone, 598 ± 25 Ma, i.e. Early Vendian.20) This is the first age constraint suggesting that the Baratal limestone formed as early as the Early Vendian.

## Study sections

We mapped at 1/1,000 scale the Kurai and Akkaya areas in the southern Gorny Altai Mountains, where the Cambrian accretionary complex is extensively exposed (Fig. 2B). It is dominated by large blocks of basaltic greenstone associated with limestone (Fig. 2B). The basaltic greenstone is composed of massive lava flows and volcaniclastic rocks. From its major and trace element composition, Utsunomiya *et al*. (1998) pointed out that the protolith basalt of the greenstone is similar to a modern mid-oceanic ridge or oceanic plateau basalt.21) Because the petrologic features of the greenstone, e.g. abundance of phenorysts of clinopyroxene, are different from those of modern mid-oceanic ridge basalt, they concluded that the greenstone was formed in a large oceanic plateau, such as Shatsky and Ontong-Java. The Baratal limestone occurs as blocks less than 200 m in diameter. The thickness of the limestone was previously estimated to be 8 km in total 16) (Fig. 2B). However, we calculate that the primary thickness before structural repetition by subduction-accretion is measured to be no greater than 500 m.

We investigated in detail four well-exposed sections KR-1, KR-2 and KR-3 in the Kurai area, and AK-1 in the Akkaya area. These sections expose a well-preserved primary depositional contact between the Baratal limestone and the underlying greenstones.

**Fig. 3:**
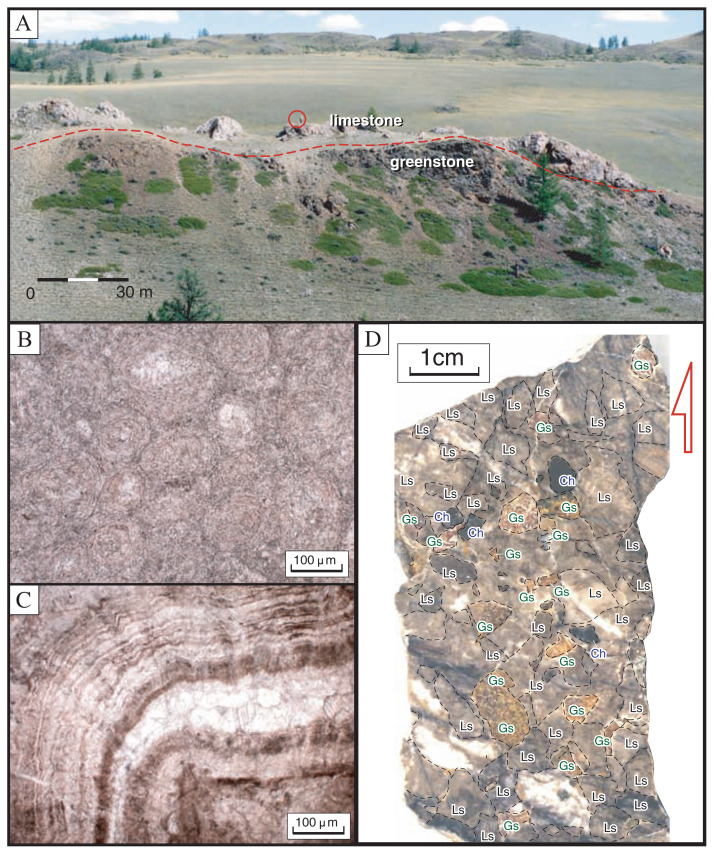
The Vendian Baratal limestone in the Kurai area, southern Siberia. A: Distant view of the KR-1 section exposing the direct contact of the Baratal limestone and the underlying basaltic greenstone. A person highlighted in a circle for scale. B: Photomicrograph of ooid packstone in the Kurai area. C: Photomicrograph of stromatolite at the KR-1 section. D: Cross section of polished limestone conglomerate/breccia at the AK-1 section. Ls: limestone, Gs: greenstone, Ch: chert.

**Fig. 4:**
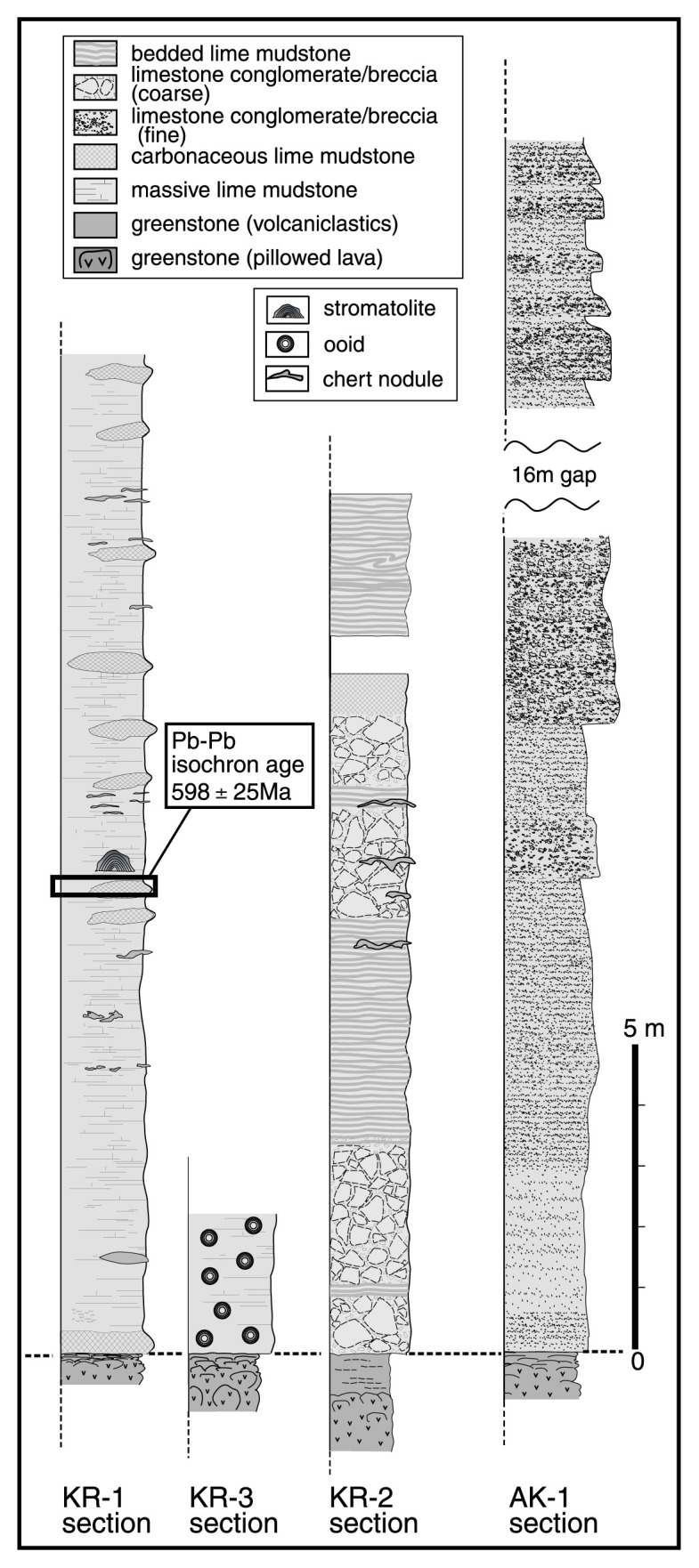
Stratigraphic columns of the basal Baratal limestone at the KR-1, KR-2, KR-3 and AK-1 section, in the Gorny Altai Mountains. Note that the Baratal limestone directly overlies the basaltic greenstone with OIB affinitiy in these 4 sections regardless of rock types of limestone. Note the bulk Pb-Pb age, 598 ± 25 Ma, measured at the KR-1 section [Nohda *et al*. (2003)].

At the KR-1 section in the Kurai area (Fig. 2B), gray, massive lime mudstone overlies directly basaltic greenstones (Fig. 3A), infilling a concave surface of the latter. The mutual contact is generally flat and parallel to the bedding of the limestone (Fig. 3A). Fig. 4 shows a 14 m-thick massive limestone immediately above the greenstone. Stromatolites occurs in a bed ca. 7 m above the limestone base. The Pb-Pb isochron age of 598 ± 25 Ma (Early Vendian) of the basal part of the Baratal limestone was measured in this section. At the KR-2 section the Baratal limestone, composed of limestone conglomerate and inter-bedded lime mudstone, overlies directly basaltic greenstone. At the KR-3 section (Fig. 2B) a gray, massive ooid packstone lies immediately above a basaltic greenstones (Fig. 4), infilling the concave top of the latter. The mutual contact is generally flat and parallel to the bedding of limestone. At the AK-1 section, greenstone is overlain by over 40 m of limestone conglomerate (Fig. 4).

These field observations indicate that the Baratal limestone in the Kurai and Akkaya areas was consistently deposited on a basement composed of oceanic plateau-type basalt.

## Lithologies of the Baratal limestone

Lithologic characteristics of the Baratal limestone are described from our field research and microscopic study of more than 300 thin sections. The limestone comprises the following 3 distinctive rock types, i.e., 1) ooid-bearing massive lime mudstone, 2) thinly bedded limestone, and 3) limestone conglomerate/breccia. These lithologies mostly consist of pure carbonate, and lack coarse-grained terrigenous (quartzo-feldspathic) clastic material.

### Ooid-bearing massive lime mudstone

This is exposed widely in the Kurai area, a representative outcrop being in the KR-1 section (Fig. 3A). The lime mudstone is composed mostly of fine-grained calcite (lime mud), with very minor thin layers of silica and carbonaceous materials. The only notable structure is unclear bedding. In some horizons ooids, 150–500 μm in diameter, amount to 50–80% by volume, giving rise to an ooid-packstone/grainstone (Fig. 3B). Stromatolites occur in 2 horizons, forming ca. 40 cm tall, 40 cm wide, upright domal structure. Banding of the stromatolites consists of a ca. 20–50 μm thick alternation of calcareous and carbonaceous laminae (Fig. 3C). Carbonaceous sphericial structures, ca. 100 μm in diameter that appear like calcareous algae occur in 3 horizons. In addition, spherical microfossils 100–500 μm in diameter with siliceous and phosphatic shells were found for the first time in the lime mudstone and the ooid-pack/grainstone, respectively; they are not yet identified nor described.

### Bedded limestone

This lithology is observed in the KR-2 and AK-1 sections. The bedded limestone is composed of a rhythmic alternation of 5–20 cm thick lime mudstone and 5–10 cm thick carbonaceous lime mudstone. In several horizons in the bedded limestone tight intraformational folds and/or boudiage indicate soft-sediment deformation. No fossils were found.

### Limestone conglomerate/breccia

This lithology occurs widely in the Akkaya and Kurai areas. It is generally massive, but locally has indistinct bedding. The conglomerate/breccia has a grain-supported and partly matrix-supported texture. Clasts range in diameter from 2 mm to 60 cm, and are mostly angular, poorly sorted, and not graded. Limestone (lime mudstone, ooid-packstone/grainstone) amounts to 85% by volume of the clasts, and is associated with minor amount of greenstone and chert clasts (Fig. 3D). The matrix of the conglomerate/breccia consists of lime mudstone. No fossils were found.

## Discussion

The direct depositional contact between the OIB-type basaltic greenstone and the Baratal limestone suggests that all lithologies of the limestone were primarily deposited on and around an ancient oceanic plateau. Absence of coarse-grained terrigenous (quartzo-feldspathic) clastics material in all types of the Baratal limestone indicates that the greenstone/limestone complex was derived from a mid-oceanic realm remote from any continents.

### Top of the paleo-plateau/seamount

The ooidbearing lime mudstone in the Kurai area may have formed in a shallow marine environment with minor influence of currents and waves, like the back reef lagoon of modern Bahama Bank.22) The occurrence of numerous ooid grains in the massive lime mudstone indicates a very shallow marine, tidal zone with strong water agitation, probably under a dry, hot climate. The occurrence of stromatolites likewise indicates a tidal to subtidal zone where cyanobacteria may conduct photosynthesis. 23) The predominance of lime mud rather than bioclasts and the framework and occurrence of stromatolites suggest that the Baratal limestone in part at least, may have been formed in a microbial reef, specifically in an agglutinated microbial reef in which particulate sediments are trapped and bound by bacteria.24)

### Slope deposits around a paleo-plateau/seamount

The indistinct bedding, poor grading/sorting of clasts, and matrix-supported texture of the limestone conglomerate/breccia are similar to those of modern debris flow deposits formed by the gravitational collapses of a slope surface, like that in the modern Bahama Bank.25) The predominance of shallow marine limestone (ooid pack-/grainstone) in angular clasts suggests that they were derived directly from an adjacent shallow-marine carbonates platform. The absence of coarse-grained quartzo-feldspathic materials both in clasts and matrix likewise suggests that the conglomerate/breccia formed in an ocean distant from a large continent. Thus, this conglomerate/breccia probably formed on slope to base-of-slope around an ancient mid-oceanic carbonate platform margins. The intimate association of the fine-grained bedded limestone and the limestone conglomerate/breccia indicates that the former also formed in a slope to base-of slope setting. The bedded limestone probably corresponds to a distal part of a limestone turbidite often developed on slopes around a carbonate platform. 25) The intraformational folds and boudinage of the bedded limestone likewise indicate unstable submarine slope environment where unconsolidated sediments are prone to slide down gravitationally as slump deposits.

**Fig. 5:**
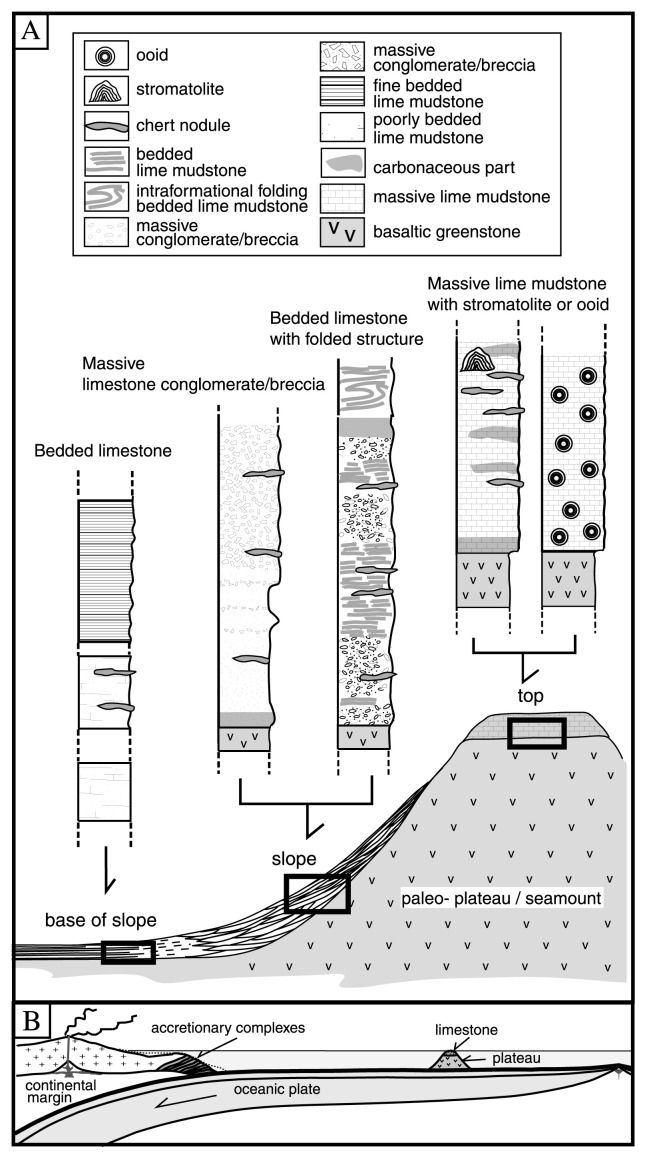
A schematic reconstruction diagram showing sedimentary setting of the three rock types of the Baratal limestone, i.e., massive limestone, bedded limestone, and limestone conglomerate (A) with a simplified ridge-trench transect (B) modified from Isozaki *et al*. (1990). The Baratal limestone was primarily deposited in a mid-oceanic realm.

The above relations suggest that the Vendian Baratal limestone in the Gorny Altai Mountains formed primarily on and around an oceanic plateau in a mid-oceanic domain. The Vendian oceanic plateau capped by the Baratal limestone moved laterally along with the underlying oceanic plate, and finally reached a circum-Siberian trench and was subducted, leaving fragments within the accretionary complex (Fig. 5B).

The depositional site of three distinctive types of the Baratal limestone can be reconstructed as shown in Fig. 5A. The ooid- and stromatolite-bearing massive lime mudstone may have deposited on the top of an ancient oceanic plateau, partly forming primitive microbial reef, while bedded limestone and limestone conglomerate/breccia accumulated on the slope to base-of-slope in gravity-induced turbidites and debris flows. Examples of such ancient mid-oceanic carbonate buildups with similar lateral facies transition were reconstructed from the allochthonous Permo-Carboniferous limestone bodies in the Late Permian accretionary complex in Southwest Japan.11),26),27)

It is noteworthy that the Vendian Baratal limestone in the Gorny Altai Mountains represents the oldest known example of an isolated platform complex on an oceanic plateau/seamount, and these rocks provide the only information on a late Neoproterozoic mid-oceanic environment immediately after the snowball Earth event28),29) and before the well-known Cambrian radiation. 2)–4) In particular, the occurrence of various microfossils with siliceous and phosphatic shells suggests that biomineralization of hard-tissued organisms had also started in a shallow mid-oceanic realm already in the Early Vendian. Details of the microfossils will be reported elsewhere.
